# Ventilation–perfusion effects of negative-pressure ventilation: insights from an experimental rat model

**DOI:** 10.1186/s40635-026-00875-8

**Published:** 2026-03-03

**Authors:** Gergely H. Fodor, Ferenc Peták, Petra Somogyi, Bence Ballók, Fruzsina Kun-Szabó, József Tolnai

**Affiliations:** 1 Department of Medical Physics and Informatics, University of Szeged, 9 Korányi Fasor, 6720 Szeged, Hungary; 2 Cerebral Blood Flow and Metabolism Research Group, Hungarian Centre of Excellence for Molecular Medicine, University of Szeged, 4 Somogyi Utca, 6720 Szeged, Hungary; 3 Department of Cell Biology and Molecular Medicine, University of Szeged, 4 Somogyi Utca, 6720 Szeged, Hungary

**Keywords:** Iron lung, Ventilation–perfusion mismatch, Ventilation dead space, Mechanical ventilation

## Abstract

**Background:**

Mechanical ventilation typically utilizes positive-pressure ventilation (PPV), which fundamentally differs from physiological pressure conditions. In contrast, negative-pressure ventilation (NPV) more closely mimics physiological pressure conditions; however, its impact on ventilation–perfusion matching remains unclear. Therefore, we compared PPV and NPV in terms of their effects on ventilation–perfusion matching and determined the consequences of increasing end-expiratory pressure (EEP).

**Methods:**

Anesthetized rats (n = 9) were ventilated using PPV at a positive EEP of 0, 3, 6, and 9 cmH_2_O. NPV was initiated by placing the rats in a sealed chamber and generating cyclic negative-pressure changes around the body while maintaining identical EEP and tidal volumes. At each EEP level, the arterial partial pressures of oxygen (PaO_2_) and CO_2_ (PaCO_2_) were measured from blood samples. Phase 2 (S2V) and 3 slopes (S3V), Fowler’s anatomical dead space fraction (VDF), and physiological dead space fractions according to Bohr (VDB) and Enghoff (VDE) were determined by volumetric capnography.

**Results:**

Higher PaO_2_ and lower PaCO_2_ were observed during NPV compared with PPV. The lower S2V and S3V values were associated with reduced VDF and VDB during NPV, whereas VDE including alveolar compartments with intrapulmonary shunt was higher. Elevating positive EEP during PPV increased S2V, S3V, and VDB, whereas the same lung expansion with NPV had a smaller effect.

**Conclusions:**

The results indicate that compared with PPV, NPV enhances gas exchange and ventilation–perfusion matching in healthy lungs. Although NPV causes fewer ventilation–perfusion inequalities and reduced dead space ventilation, its efficacy may be limited by increased intrapulmonary shunting during excessive negative end-expiratory pressure levels. These results provide mechanistic support for the physiological benefits of subatmospheric ventilation and may provide a basis for further studies on the refinement of noninvasive and lung-protective ventilation strategies in clinical settings with impaired ventilation–perfusion matching, such as acute respiratory failure, postoperative care, and ventilator weaning.

## Introduction

Lung ventilation is governed by cyclic alterations in the driving pressure during breathing cycles. In spontaneous breathing, the expansion of the thoracic cavity generates subatmospheric (negative) pressure in the alveoli during inspiration. This results in an influx of air into the gas exchange zone of the lungs along the pressure gradient. In contrast, mechanical ventilation, which is often used under general anesthesia and intensive care, applies positive pressure to the airway opening to facilitate inspiration by forcing air into the alveoli. The pressure conditions during positive-pressure ventilation (PPV) differ fundamentally from physiological breathing, which presents significant challenges for healthcare providers. These include the risk of ventilator-induced lung injury (VILI) [1–3] and compromised cardiac output due to elevated intrathoracic pressure [4–6]. In addition, these detrimental effects may be exacerbated by adverse changes in pulmonary hemodynamics, such as pulmonary capillary derecruitment and increased pulmonary vascular resistance (PVR), when positive-pressure lung expansion occurs [7–9].

Mechanical ventilation using subatmospheric extrathoracic pressure was established during the early days of modern intensive therapy. Negative pressure ventilation (NPV) provides more physiological pressure conditions in the respiratory system. This explains the beneficial characteristics of NPV, including improved gas exchange [10, 11] and the prevention of VILI [12]. In addition, because intrathoracic pressure during NPV is similar to spontaneous breathing, the adverse hemodynamic effects often observed with PPV do not occur [7, 13–17]. Thus, NPV maintains cardiac output and improved lung perfusion [7].

Volumetric capnography provides a noninvasive assessment of ventilation–perfusion (V/Q) matching. The phase 2 slope (S2V) reflects the transition between conducting airways and alveolar compartments, whereas the phase 3 slope (S3V) reflects the homogeneity of alveolar V/Q ratios and perfusion distribution. Together with the derived ventilation dead space indices, these shape factors allow mechanistic discrimination between ventilation inefficiency, perfusion impairment, and intrapulmonary shunt, making volumetric capnography a particularly suitable tool to investigate alterations in V/Q matching.

In clinical practice, the revival of negative-pressure ventilation with cuirass- and shell-based devices has renewed interest in its potential advantages for patients requiring noninvasive or lung-protective ventilation, such as during ventilator weaning, in patients with neuromuscular disorders, or for respiratory support following extubation [18–20]. Understanding how the pressure environment influences V/Q matching at the organ level could help in optimization of ventilatory support.

Despite the contrasting effects of PPV and NPV on lung perfusion, the differences between these modalities in terms of V/Q matching have not been adequately compared. Specifically, how pressure-induced changes in capillary recruitment, airway geometry, and perfusion heterogeneity translate into effective ventilation is unclear, necessitating approaches that assess V/Q relationships beyond global gas exchange indices. Therefore, we compared the parameters that reflect V/Q matching during PPV and NPV by applying volumetric capnography to determine whether the end-expiratory pressure (EEP) influences this relationship.

## Methods

### Ethical approval

The protocol for this study was approved by the National Food Chain Safety and Animal Health Directorate of Csongrád County, Hungary (no. XXXIII./2366/2023) on December 4, 2023. The study complied with the guidelines of the Scientific Committee of Animal Experimentation of the Hungarian Academy of Sciences (updated Law and Regulations on Animal Protection: 40/2013. [II. 14.], the Government of Hungary) and the European Union Directive 2010/63/EU for the protection of animals used for scientific purposes. The methods and results were reported in accordance with the ARRIVE guidelines [21].

### Animal preparations

Nine Sprague–Dawley rats (244–402 g, CD^®^ IGS Rat, Charles River, Germany, 5 males, 4 females) were included in the protocol. The animals were anesthetized with an intraperitoneal injection of sodium pentobarbital (45 mg/kg i.p.) [22, 23]. They were then placed in a supine position on a heating pad, with a rectal thermometer connected and set to 37.0 °C ± 0.5 °C (Model 507223F, Harvard Apparatus, South Natick, MA, USA). Following the subcutaneous application of lidocaine (2–4 mg/kg), tracheostomy was performed using a 2.5-mm metal cannula (outer diameter 2.5 mm, cat.no. #732,725, Harvard Apparatus, South Natick, MA, USA). The animals were mechanically ventilated with PPV using a rodent ventilator (tidal volume: 10 ml/kg [24, 25], room air, positive end-expiratory pressure of 3 cmH_2_O, frequency to achieve normocapnia at 60–70 BPM, Model 683, Harvard Apparatus, South Natick, MA, USA). The dynamics of expired CO_2_ were assessed using a sidestream rodent capnograph (Type 340, Harvard Apparatus, South Natick, MA, USA). The left femoral artery and vein were cannulated with polyethylene tubes (Abbocath 22G). Blood samples were drawn from the femoral artery for arterial blood gas measurements (epoc® Reader and Host, Epocal Inc., Ottawa, ON, Canada), and a femoral venous line was also secured for drug delivery. Following preparation of the vessels, anesthesia was maintained by repeated intravenous injections of sodium pentobarbital (12 mg/kg, every 30 min) [22, 23]. Ventilation waveforms were monitored and recorded using a data collection and acquisition system (PowerLab 8/35 and LabChart, ADInstruments, Dunedin, New Zealand). The heating pad was removed at the end of preparation.

### Application of PPV and NPV

**Fig. 1:**
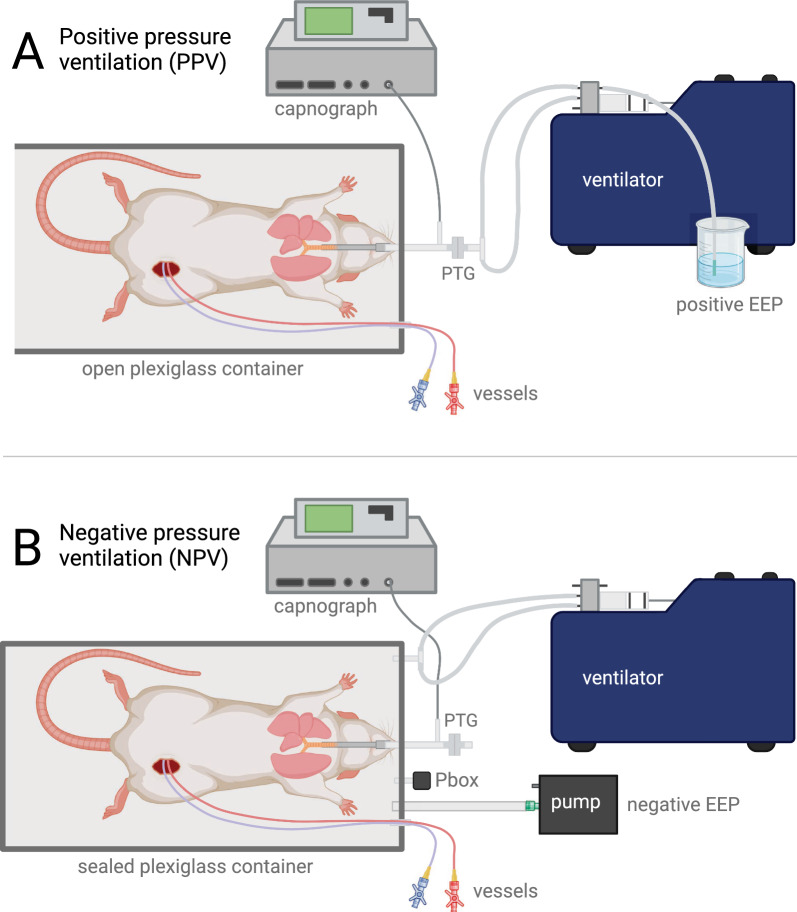
Scheme of the experimental setup. The top panel (**A**) demonstrates the equipment used for positive-pressure ventilation (PPV); the bottom panel (**B**) illustrates negative-pressure ventilation (NPV) setup. During PPV, a water-filled container was used to generate various levels of positive end-expiratory pressure (EEP), while an adjustable pump was used to generate negative EEP during NPV. Further details of the setup are described in the text. *PTG* pneumotachograph for measuring respiratory airflow, *Pbox* measurement of pressure inside the sealed container relative to atmospheric pressure

The scheme of the experimental setup is illustrated in Fig. 1. Positive-pressure ventilation was applied using a volume-controlled rodent ventilator (Model 683, Harvard Apparatus, South Natick, MA, USA) connected to the trachea. Ventilation was carried out using room air, a tidal volume of 10 ml/kg, and a frequency adjusted to achieve normocapnia. The ventilatory flow was monitored by a small animal heated screen pneumotachograph (Model 8430, Hans Rudolph Inc., Shawnee, KS, USA) and a differential pressure transducer (24PCEFA6D, Honeywell, Charlotte, NC, USA). Positive end-expiratory pressure (positive EEP) was achieved by submerging the exhaust port of the ventilator into a water-filled column.

Negative pressure ventilation was achieved by moving the air inside a sealed plexiglass container (volume = 1.91 L, with a water-filled rubber glove added inside to reduce dead space) using the same type of volume-controlled rodent ventilator equipped with a 20-ml piston. The fresh gas supply and inspiratory ports of the ventilator were connected to the plexiglass container, which resulted in rhythmic decreases and returns of box pressure leading to expansion and retraction of the chest and consequent breathing. The box pressure was monitored with a differential pressure transducer relative to room air (MPX5010DP, NXP Semiconductors, Eindhoven, The Netherlands). The tidal volume of the ventilator was adjusted, so the ventilatory tidal volume measured at the tracheal opening matched the volume measured during PPV. An adjustable pump (CO2100C, Biopac Systems Inc., Goleta, CA, USA) was attached to the container to maintain a constant end-expiratory pressure. The pump speed was adjusted manually using a screw potentiometer to achieve and maintain the desired negative EEP inside the container. The trachea was connected only to the sampling port of the capnometer and the pneumotachograph, without an added ventilator.

To minimize the differences between the experimental setups established for the two ventilation modes, the animals were placed in a plexiglass container after the preparation was completed, with the container open. The tracheal cannula and the arterial and venous catheters were connected to the ports of the container and passed through to the outside. The sampling site for the capnograph and the pneumotachograph were connected to the tracheal port from outside the container. The Y-piece of the PPV circuit was attached to the distal end of the pneumotachograph during PPV and removed during NPV.

### Experimental protocol

Once the animals were placed into the experimental setup, PPV was initiated with an EEP of 0 cmH_2_O. To normalize volume history, a single recruitment maneuver was done at EEP of 0 cmH_2_O by temporarily blocking the exhaust port of the ventilator, resulting in a single double-volume breath, followed by standard tidal volume breaths [26, 27]. Following a 3-min equilibration period, volumetric capnography was recorded for 1 min and an arterial blood gas sample was subsequently analyzed. Positive EEP was increased to + 3, + 6, and + 9 cmH_2_O, and the same procedure was repeated for each EEP, i.e., after the increase of EEP and a 3-min equilibration period capnography was recorded, and arterial blood gas sample was analyzed. EEP was then dropped to 0 cmH_2_O, the container was closed, and NPV was initiated at 0 EEP. During NPV, the Y-piece of the PPV ventilation circuit was disconnected. The pump speed and box ventilation were adjusted to correct the ventilatory volume. After a 3-min equilibration period, volumetric capnography was recorded for 1 min, and an arterial blood gas sample was analyzed. The pump speed was adjusted to achieve negative EEP levels of -3, -6, and -9 cmH_2_O and the box ventilation was adjusted to match the tidal volume of the positive EEP counterpart (10 ml/kg tidal volume) with a maximum of 10% tolerance [28]. In case EEP deviated from the set level by more than 0.1 cmH_2_O in either direction, pump speed was adjusted to restore EEP. The same procedure was performed for each negative EEP level.

The selected EEP levels (0, ± 3, ± 6, and ± 9 cmH_2_O) were chosen to span a physiologically and clinically relevant range of lung volumes while avoiding extremes associated with lung injury or derecruitment. In small rodents, these pressures result in similar amount of lung tissue strain as those produced by commonly applied PEEP levels in humans [29].

### *Assessment of exhaled CO*_*2*_* dynamics*

The dynamics of expired CO_2_ were recorded using a sidestream rodent capnograph (Harvard Capnograph Type 340) and tracheal airflow was measured with a screen pneumotachograph (Model 8430, Hans Rudolph Inc., Shawnee, KS, USA). Both signals were digitized at a sampling rate of 256 Hz and analyzed using custom-developed software.

Volumetric capnography was obtained by combining the expiratory CO_2_ signals and the volume signals obtained by integrating tracheal airflow. Time domain capnogram recordings were offset with a constant of −0.58 s for PPV and −0.61 s for NPV relative to the flow signals to adjust for the transport delay caused by gas being drawn into the analyzer. These delay constants were determined by using a step-change in CO_2_ from a 5% CO_2_ cylinder and examining the delay between the onset of flow and the capnogram increases [30].

The assessment of the shape indices was based on the concepts described earlier [31, 32]. Briefly, the phase 2 slopes (S2V), which characterize the interface between the airway and alveolar compartments, were calculated by determining the slope at the inflection point. The phase 3 slopes of the volumetric capnograms (S3V), dominated by the alveolar compartment, were defined by linear regression fitted to the middle third of phase 3. Because the magnitudes of the slopes of the capnogram curves are affected by the end-tidal CO_2_ concentration (ETCO_2_), these slopes were also expressed as normalized values by dividing the slopes with the respective ETCO_2_ levels (Sn2V and Sn3V for normalized phase 2 and phase 3 slopes, respectively).

Since volumetric data were obtained, the ventilation dead space parameters were also calculated after correcting for the instrumental dead spaces (1.14 ml for PPV and 0.70 ml for NPV). The anatomical dead space (according to Fowler; VDF) was defined as the volume exhaled up to the inflection point of phase 2 [33]. The physiological dead space according to Bohr (VDB) was calculated as follows [34]:

$${\mathrm{VDB}}\,/{\mathrm{V}}_{{\mathrm{T}}} \, = \,({\mathrm{PACO}}_{{2}} - {\mathrm{P}}\overline{{\mathrm{E}}} {\mathrm{CO}}_{{2}} )/{\mathrm{PACO}}_{2} ,$$

where PACO_2_ is the mean alveolar partial pressure of CO_2_, obtained using the value at the midpoint of phase 3 in the volumetric capnograms. PĒCO_2_ is the mixed expired CO_2_ partial pressure value, which was determined for each expiratory cycle by dividing the integrated volumetric capnogram curve by the current expired gas volume (V_T_).

Physiological dead space was calculated according to Enghoff’s modification (VDE; which also includes intrapulmonary shunt) as follows [35]:

$${\mathrm{VDE/V}}_{{\mathrm{T}}} \, = \,({\mathrm{PaCO}}_{{2}} {-}{\mathrm{P}}\overline{{\mathrm{E}}} {\mathrm{CO}}_{2} )/{\mathrm{PaCO}}_{{2}} ,$$

where PaCO_2_ is the partial pressure of CO_2_ in the arterial blood sample.

The dead spaces according to Fowler, Bohr, and Enghoff are expressed as fractions of tidal volume by dividing the corresponding dead space volumes by V_T_. The difference between Enghoff’s and Bohr’s dead spaces represents the intrapulmonary shunt circulation (i.e., a virtual lung volume corresponding to the perfused, but not ventilated, alveoli). The arterial-alveolar CO_2_ gradient (P_a-A_CO_2_) as a further measure of intrapulmonary shunt was calculated as the difference between PaCO_2_ and PACO_2_.

### Statistical analyses

Sample size estimation was based on a two-way repeated-measures analysis of variance (ANOVA) design, assuming an effect size of 0.4, a power of 0.8, and a significance level of 0.05. This indicated that at least eight animals were required to detect a statistically significant difference [36]. The estimations were carried out using the software G*Power (version 3.1.9.7, Universität Düsseldorf, Germany).

Data are expressed as mean ± standard deviation (SD). Normality was assessed using the Shapiro–Wilk test, and two-way repeated-measures ANOVA was done to assess the changes in capnography indices using the mode of ventilation (PPV or NPV) and EEP levels (0, ± 3, ± 6, or ± 9 cmH_2_O) as factors for comparison. Pairwise comparisons were performed by the Holm–Sidak post hoc test. Statistical analyses were conducted at a significance level of p < 0.05. Effect sizes are reported as model-derived mean differences between negative- and positive-pressure ventilation (NPV–PPV) with 95% confidence intervals, averaged across end-expiratory pressure levels and calculated using Kenward–Roger degrees of freedom.

## Results

**Fig. 2:**
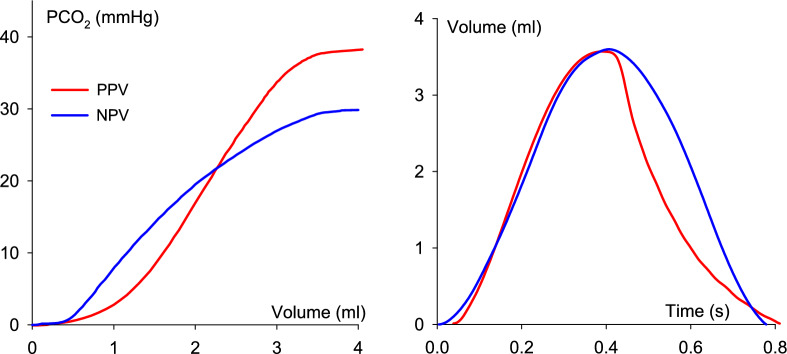
Representative volumetric capnogram and ventilatory volume curves obtained in the same animal at the same end-expiratory pressure levels during positive-pressure ventilation (PPV, red curves) and negative-pressure ventilation (NPV, blue curves). *PCO*_*2*_ partial pressure of exhaled carbon dioxide

The volumetric capnogram curves obtained from a representative animal during both PPV and NPV are shown in Fig. 2 (left panel). Although both capnograms exhibit characteristic shapes, there were apparent differences in the ETCO_2_ and slopes depending on the ventilation mode, even when identical VTs were applied. In addition, the volume patterns were affected by the ventilation mode because of the passive expiration during PPV and controlled exhalation during NPV (right panel).

**Fig. 3:**
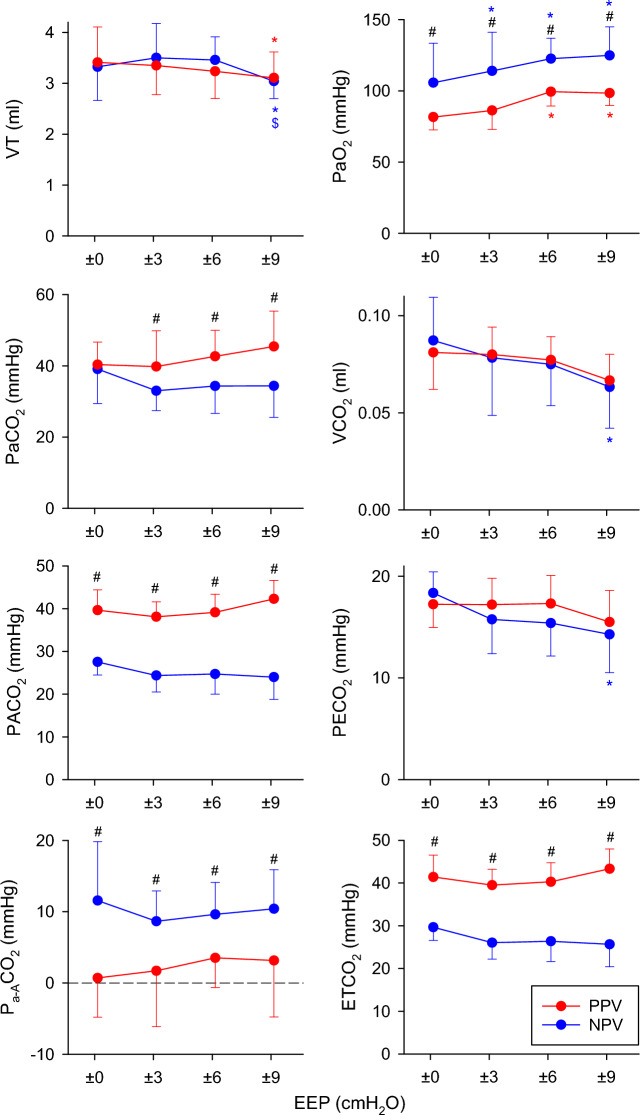
Gas exchange parameters determined by arterial blood gas analyses and volumetric capnography during positive-pressure ventilation (PPV, red curves) and negative-pressure ventilation (NPV, blue curves) at different positive or negative end-expiratory pressure (EEP) levels. *VT* tidal volume, *PaO*_*2*_ partial pressure of oxygen in the arterial blood, *PaCO*_*2*_ partial pressure of carbon dioxide in the arterial blood, *VCO*_*2*_ volume of exhaled carbon dioxide per breath, *PACO*_*2*_ mean alveolar partial pressure of carbon dioxide, *PECO*_*2*_ mixed expired partial pressure of carbon dioxide, *P*_*a-A*_*CO*_*2*_ arterial–alveolar CO_2_ difference, *ETCO*_*2*_ partial pressure of end-tidal carbon dioxide. #: p < 0.05 for NPV vs PPV at the same EEP level; *: p < 0.05 versus an EEP level of 0 cmH_2_O using the same ventilation mode; $: p < 0.05 versus an EEP level of 3 cmH_2_O using the same ventilation mode. Effect sizes comparing NPV with PPV are reported as model-derived mean differences (NPV–PPV) with 95% confidence intervals, averaged across EEP levels: VT: 0.05 ml (−0.06 to 0.17); PaO_2_: 28.4 mmHg (20.4 to 36.4); PaCO_2_: −6.9 mmHg (−10.5 to −3.2); VCO_2_: −0.000 ml (−0.007 to 0.006); PACO_2_: −14.7 mmHg (−16.9 to −12.5); PECO_2_: −0.85 mmHg (−2.39 to 0.64); P_a-A_CO_2_: 7.8 mmHg (5.1 to 10.4); ETCO_2_: −14.2 mmHg (−16.4 to −12.0)

The ventilation and gas exchange parameters during PPV and NPV at different EEP levels are summarized in Fig. 3. Changing the EEP did not affect these outcomes, with only slight changes observed in VT, PaO_2_, VCO_2_, and PECO_2_ (p < 0.05 for all). The ventilation mode had no significant effect on VT, VCO_2_, and PECO_2_; however, ventilation with NPV using identical VT and frequency yielded a higher PaO_2_ and P_a-A_CO_2_ and lower PaCO_2_ at all EEP levels (p < 0.01 for all), except for PaCO_2_ at the lowest EEP. In addition, lower PACO_2_ and ETCO_2_ were observed under NPV, regardless of the EEP used (*p* < 0.001 for all).

**Fig. 4:**
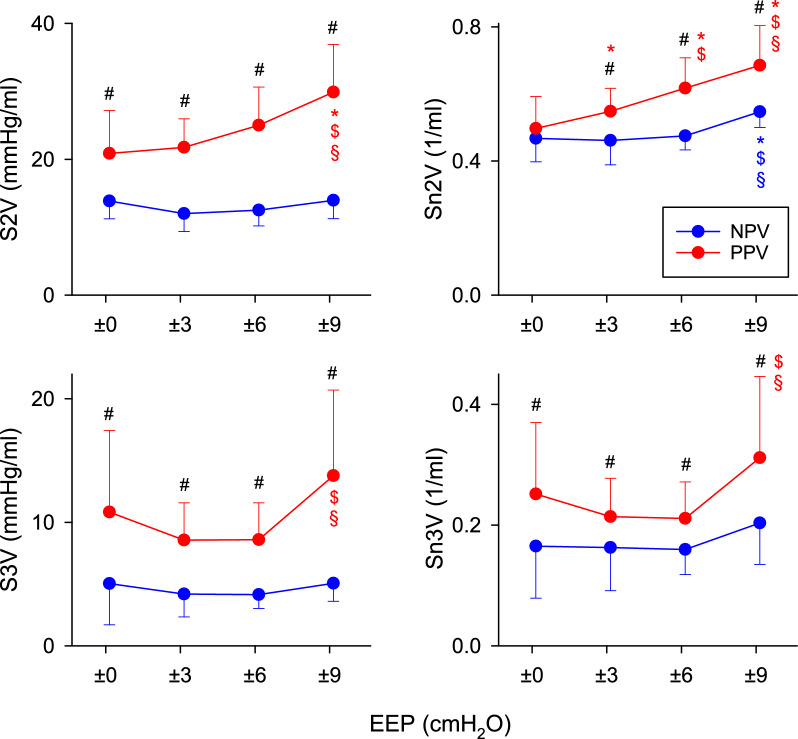
Volumetric capnography shape factors during positive-pressure ventilation (PPV, red curves) and negative-pressure ventilation (NPV, blue curves) at different positive or negative end-expiratory pressure (EEP) levels. S2V: the slope of phase 2 of the volumetric capnogram; Sn2V: the normalized slope of phase 2 of the volumetric capnogram; S3V: the slope of phase 3 of the volumetric capnogram; Sn3V: the normalized slope of phase 3 of the volumetric capnogram. #: p < 0.05 for NPV vs PPV at the same EEP level; *: p < 0.05 versus an EEP level of 0 cmH_2_O using the same ventilation mode; $: p < 0.05 versus an EEP level of 3 cmH_2_O using the same ventilation mode; §: p < 0.05 versus an EEP level of 6 cmH_2_O using the same ventilation mode. Effect sizes comparing NPV with PPV are reported as model-derived mean differences (NPV–PPV) with 95% confidence intervals, averaged across EEP levels: S2V: -11.3 mmHg/ml (−13.4 to −9.2); Sn2V: -0.10 1/ml (−0.12 to −0.07); S3V: −5.8 mmHg/ml (−7.4 to −4.3); Sn3V: −0.07 1/ml (−0.10 to −0.05)

Absolute values of the shape factors and their normalized values to ETCO_2_ derived from the volumetric capnogram curves during PPV and NPV at different EEP levels are shown in Fig. 4. The use of NPV resulted in lower S2V and S3V at all EEP levels (p < 0.01 for all). These differences were still apparent after normalizing the slopes to ETCO_2_ (p < 0.05 for all). Increasing the magnitude of EEP elevated S2V, S3V, and Sn3V under PPV at the highest EEP (p < 0.05 for all); however, these parameters remained constant under NPV.

**Fig. 5:**
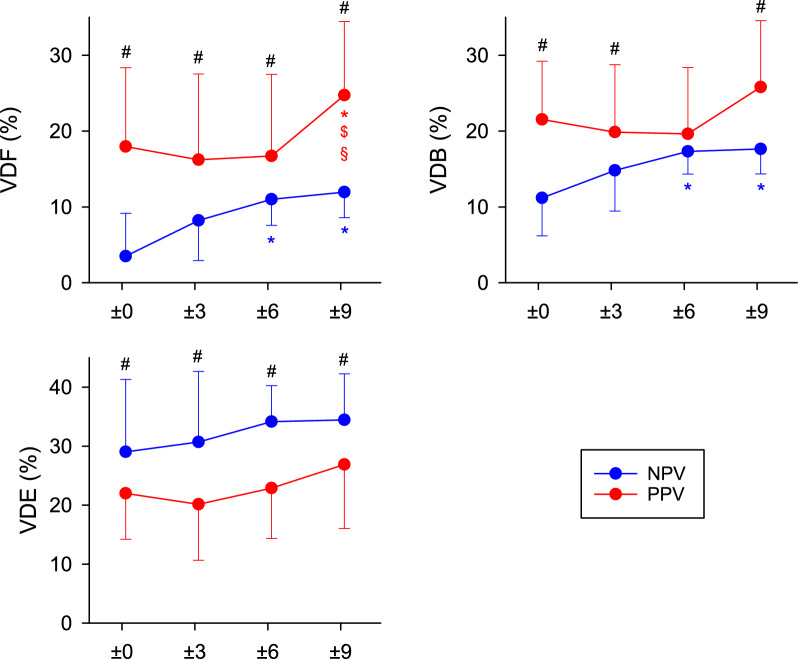
Ventilation dead space fractions obtained by volumetric capnography during positive-pressure ventilation (PPV, red curves) and negative-pressure ventilation (NPV, blue curves) at different positive or negative end-expiratory pressure (EEP) levels. *VDF* anatomical dead space fraction according to Fowler, *VDB* physiological dead space fraction according to Bohr; *VDE* physiological dead space fraction according to Enghoff. #: p < 0.05 for NPV vs PPV at the same EEP level; *: p < 0.05 versus an EEP level of 0 cmH_2_O using the same ventilation mode; $: p < 0.05 versus an EEP level of 3 cmH_2_O using the same ventilation mode. Effect sizes comparing NPV with PPV are reported as model-derived mean differences (NPV–PPV) with 95% confidence intervals, averaged across EEP levels: VDF: −17.5% (−21.4 to −13.5); VDB: −13.7% (−17.3 to −10.1); VDE: 5.5% (3.6 to 10.7)

The ventilation dead space fractions corrected for instrumental dead space measured under PPV and NPV are shown in Fig. 5. The application of NPV with the same VT decreased VDF and VDB independent of the EEP (p < 0.05 for all) similar to S2V and S3V. These reductions were associated with increases in VDE (p < 0.05).

## Discussion

This study revealed fundamental differences in matching ventilation to pulmonary perfusion and its dependence on end-expiratory pressure during mechanical ventilation. The application of subatmospheric extrathoracic pressure during mechanical ventilation resulted in better gas exchange, lower phase 2 and 3 slopes of the volumetric capnogram, and lower Fowler’s anatomic and Bohr’s physiological ventilation dead space fractions compared with positive-pressure ventilation using the same tidal volume and ventilation frequency. In contrast, Enghoff’s modified physiologic dead space fraction, which includes alveolar compartments with an intrapulmonary shunt was higher during negative-pressure ventilation. In terms of the effects of different end-expiratory pressures, their elevation during positive-pressure ventilation increased the capnography parameters reflecting ventilation–perfusion mismatch, whereas these changes were not observed during negative-pressure ventilation.

We observed improved gas exchange parameters during NPV compared to those obtained under PPV when matched VT and ventilation frequency was applied. This benefit was apparent in the arterial blood gas (PaO_2_, PaCO_2_) and capnography parameters (PACO_2_ and ETCO_2_) at all applied EEP levels, consistent with a shift toward more favorable V/Q relationships rather than simple changes in minute ventilation.

Previous studies comparing PPV and NPV have reported inconsistent effects on oxygenation, largely depending on experimental conditions and lung volume history [37, 38]. When lung recruitment and volume history were carefully matched, differences in PaO_2_ were markedly reduced [39], suggesting that some reported oxygenation benefits of NPV reflect volume history rather than pressure mode. In the present study, we minimized differences in volume history by applying recruitment maneuvers and matching tidal volume, ventilation frequency, and end-expiratory pressure magnitude between ventilation modes. Despite this, NPV consistently improved capnography-derived indices of V/Q matching across all EEP levels, likely because volumetric capnography captures functional ventilation and perfusion characteristics not fully reflected by oxygenation alone. Supporting this interpretation, recent imaging and modeling studies demonstrate distinct regional stress, strain, and perfusion patterns between PPV and NPV despite similar global lung volumes [37, 38].

The results obtained by volumetric capnography provide insight into the observed gas exchange differences between PPV and NPV. Phase 3 slope of the volumetric capnogram reflects V/Q matching, whereas Bohr’s physiological dead space also quantifies the volume of the ventilated but hypo- or underperfused alveolar compartments. Thus, the lower S3V, Sn3V, and VDB during NPV indicate improved V/Q matching compared with PPV. Because the major difference between PPV and NPV is their effect on the pulmonary vasculature [7, 40], improved pulmonary perfusion may be primarily responsible for this benefit of NPV by enabling optimal matching of perfusion to ventilation. Indeed, it has been previously established that positive-pressure lung expansion increases PVR [8, 9, 41–45]. This phenomenon results from the compression of the intraalveolar capillaries, in addition to the elongation of intra- and extraalveolar vessels [8, 9, 43–45]. In contrast, NPV exerts radial traction on the intraalveolar capillaries, thereby decreasing PVR and improving pulmonary perfusion. In addition to the pulmonary vascular effect, the negative pressure around the conducting airways during NPV may have increased their dimensions, as evidenced by the lower anatomical dead space fraction according to Fowler. This is also supported by the previous data in ex vivo sheep lung undergoing NPV, where medium and small airway caliber has been significantly increased by NPV [46]. This suggests improved ventilation during NPV as a contributing factor to the improved gas exchange. Interestingly, the modified assessment of the physiological dead space by Enghoff, which includes alveolar compartments with an intrapulmonary shunt, was higher during NPV [47]. The somewhat elevated intrapulmonary shunt with NPV was further supported by the higher P_a-A_CO_2_ levels. This seemingly controversial result may be explained by the more pronounced beneficial effect of NPV on capillary perfusion than on alveolar ventilation. Accordingly, this augmented intrapulmonary shunt suggests that during NPV, improvements in capillary perfusion may have occurred in alveolar compartments without a ventilation benefit.

With respect to the effects of different EEP levels, the ventilation mode had no major impact on the EEP-dependent changes in the gas exchange parameters. However, the mechanisms by which EEP influenced V/Q matching differed fundamentally between PPV and NPV. VCO_2_ and PECO_2_ only differed between PPV and NPV at the highest EEP. Decreases in these parameters without changes in PACO_2_, PaCO_2_, and ETCO_2_ may have resulted from a slight increase in the intrapulmonary shunt during NPV. In contrast, shape factors and dead space fractions exhibited marked differences in their EEP-dependence between PPV and NPV. The elevations in S3V, Sn3V, and VDB during PPV may be explained by the compromised alveolar perfusion following high positive EEP. This exaggerates the compression of the alveolar capillaries, thereby leading to the V/Q mismatch by leading to an increased fraction of ventilated but underperfused alveoli, explaining the observed rise in dead space indices and capnogram slopes at higher positive EEP levels. This is consistent with the results obtained previously in healthy pigs [48] and in postoperative cardiac patients ventilated with various positive end-expiratory pressures [49]. However, during NPV, this detrimental effect of the elevated effect of excessive lung expansion by negative EEP was not manifested. This may be attributed to the ability of NPV with more negative EEP to counteract the pulmonary circulatory impairment observed with PPV [7]. Thus, the development of on excessive intrapulmonary shunt limits the excessive application of EEP during NPV for optimizing gas exchange outcomes.

Translationally, our observations emphasize that restoring a more physiological pressure environment similar to spontaneous breathing can favorably influence perfusion distribution even in the absence of parenchymal injury. This can be particularly relevant for the development of noninvasive NPV devices in patients unable to sustain spontaneous breathing or in those where minimizing intrathoracic pressure is desired to protect cardiac function.

Our study requires a few methodological considerations. The same tidal volumes and frequencies at identical but opposite sign EEP levels were delivered during both ventilation modes. Although the volume patterns were identical during inspiration, there was a difference in the expiratory flow, and therefore, the volume curves between NPV and PPV (Fig. 2, right panel). During expiration, passive elastic recoil of the chest and the lung tissues resulted in a typical rapid decelerating flow during PPV, which led to a rapid decrease in gas volume during the early phase of expiration. In contrast, the rate of change in the expired volume during NPV was slower, because the rate of change of the expired volume was controlled by the ventilator. Although this methodological difference could have distorted the time capnogram, expressing the changes in CO_2_ against the expired volume reduces sensitivity to flow-dependent distortions and provides a more robust assessment of ventilation–perfusion relationships. The controlled expiratory flow pattern under NPV resembles flow-controlled ventilation. Such flow pattern is known to promote earlier alveolar gas contribution and improved convective–diffusive gas mixing, which can shift the phase 2 inflection toward lower expired volumes and thereby reduce Fowler dead space [50, 51]. Nevertheless, the differences in the flow pattern may partly also contribute to the effects of NPV on VDE, as higher levels of intrapulmonary shunt were reported previously using delayed expiration compared with conventional PPV [52]; however, it may be expected that NPV with a similar passive exhalation and decelerating flow pattern can have further benefits without increasing the intrapulmonary shunt. However, the consistent direction and magnitude of differences between PPV and NPV across all EEP levels, together with concordant changes in arterial blood gases, support the physiological relevance of the findings rather than a consequence of a methodological difference. In addition, pulmonary perfusion was inferred indirectly from gas exchange and capnography indices, as no direct perfusion imaging was performed.

Another limitation of the study relates to the exposure area to negative pressure. In the present setup, subatmospheric pressure was applied to the whole body of the rat, similar to iron lung ventilation [53]; however, negative-pressure ventilators recently applied subatmospheric pressure only around the thorax using various methods, such as cuirass shells [54, 55]. Whole body exposure may have affected systemic hemodynamics and blood distribution, because all extrathoracic areas were expanded by the subatmospheric pressure, not only the thoracic cavity and the surrounding vessels [56]. Consequently, the hemodynamic and V/Q benefits observed here may represent a conservative estimate compared with methods using a cuirass shell around the chest [15]. These differences should be considered when translating the present findings into clinical practice, and warrant further studies comparing volumetric capnography outcomes during whole-body and limited chest negative-pressure ventilation. Moreover, airway and transpulmonary pressures were not directly measured during NPV, limiting direct quantification of transpulmonary stress under subatmospheric conditions.

Since the small size of the respiratory system of rats prohibits the use of mainstream capnography sensors, a further methodological consideration involves the use of sidestream volumetric capnography. Sidestream assessments of parameters associated with the late phase of expiration (S3V, Sn3V, VDB, and VDE) are reliable and accurate [30]; however, distal gas analysis by sidestream capnography can bias the absolute values of the parameters associated with the early phase of expiration (S2V, Sn2V, and VDF) [30, 57, 58]. Nonetheless, the significant correlations with mainstream outcomes suggest that comparisons of NPV and PPV are valid even for these indices [30].

While the study was conducted in healthy rats, the controlled design allows isolation of the pure mechanical effects of pressure mode on V/Q matching. Future studies in disease models and large-animal settings are needed to confirm whether the same physiological advantages persist under clinically relevant conditions such as ARDS, pulmonary edema, or restrictive lung disease.

## Conclusions

In summary, negative-pressure ventilation improved gas exchange and ventilation–perfusion matching compared with positive-pressure ventilation under identical tidal volume and frequency. These benefits were reflected by reduced ventilation dead space and more favorable capnography indices. However, at excessive negative end-expiratory pressures, intrapulmonary shunting increased, suggesting that the physiological advantage of negative-pressure ventilation is limited by augmented shunting rather than impaired perfusion. Together, these findings offer physiological evidence for the potential use of negative-pressure ventilation as a lung-protective strategy when applied within an appropriate pressure range.

## Data Availability

The datasets used and/or analyzed during the current study are available from the corresponding author on reasonable request.

## Abbreviations

EEP: End-expiratory pressure
ETCO_2_: End-tidal CO_2_ concentration
NPV: Negative pressure ventilation
PaO_2_: Arterial partial pressure of oxygen
PaCO_2_: Arterial partial pressure of carbon dioxide
PACO_2_: Mean alveolar partial pressure of CO_2_
P_a-A_CO_2_: Arterial-alveolar CO_2_ gradient
PĒCO_2_: Mixed expired CO_2_ partial pressure
PPV: Positive-pressure ventilation
PVR: Pulmonary vascular resistance
S2V: Phase 2 slope of volumetric capnogram
S3V: Phase 3 slope of volumetric capnogram
Sn2V: Normalized phase 2 slope of volumetric capnogram
Sn3V: Normalized phase 3 slope of volumetric capnogram
VDB: Ventilation dead space fraction according to Bohr
VDE: Ventilation dead space fraction according to Enghoff
VDF: Ventilation dead space fraction according to Fowler
VILI: Ventilator-induced lung injury
V/Q: Ventilation–perfusion ratio
VT: Tidal volume
